# Characterising the grey matter correlates of leukoaraiosis in cerebral small vessel disease

**DOI:** 10.1016/j.nicl.2015.07.002

**Published:** 2015-08-13

**Authors:** Christian Lambert, Janakan Sam Narean, Philip Benjamin, Eva Zeestraten, Thomas R. Barrick, Hugh S. Markus

**Affiliations:** a Neurosciences Research Centre, Cardiovascular and Cell Sciences Research Institute, St George's University of London, United Kingdom; b Stroke Research Group, Division of Clinical Neurosciences, University of Cambridge, United Kingdom

## Abstract

Cerebral small vessel disease (SVD) is a heterogeneous group of pathological disorders that affect the small vessels of the brain and are an important cause of cognitive impairment. The ischaemic consequences of this disease can be detected using MRI, and include white matter hyperintensities (WMH), lacunar infarcts and microhaemorrhages. The relationship between SVD disease severity, as defined by WMH volume, in sporadic age-related SVD and cortical thickness has not been well defined. However, regional cortical thickness change would be expected due to associated phenomena such as underlying ischaemic white matter damage, and the observation that widespread cortical thinning is observed in the related genetic condition CADASIL (Righart et al., 2013).

Using MRI data, we have developed a semi-automated processing pipeline for the anatomical analysis of individuals with cerebral small vessel disease and applied it cross-sectionally to 121 subjects diagnosed with this condition. Using a novel combined automated white matter lesion segmentation algorithm and lesion repair step, highly accurate warping to a group average template was achieved. The volume of white matter affected by WMH was calculated, and used as a covariate of interest in a voxel-based morphometry and voxel-based cortical thickness analysis. Additionally, Gaussian Process Regression (GPR) was used to assess if the severity of SVD, measured by WMH volume, could be predicted from the morphometry and cortical thickness measures.

We found significant (Family Wise Error corrected p < 0.05) volumetric decline with increasing lesion load predominately in the parietal lobes, anterior insula and caudate nuclei bilaterally. Widespread significant cortical thinning was found bilaterally in the dorsolateral prefrontal, parietal and posterio-superior temporal cortices. These represent distinctive patterns of cortical thinning and volumetric reduction compared to ageing effects in the same cohort, which exhibited greater changes in the occipital and sensorimotor cortices. Using GPR, the absolute WMH volume could be significantly estimated from the grey matter density and cortical thickness maps (Pearson's coefficients 0.80 and 0.75 respectively).

We demonstrate that SVD severity is associated with regional cortical thinning. Furthermore a quantitative measure of SVD severity (WMH volume) can be predicted from grey matter measures, supporting an association between white and grey matter damage. The pattern of cortical thinning and volumetric decline is distinctive for SVD severity compared to ageing. These results, taken together, suggest that there is a phenotypic pattern of atrophy associated with SVD severity.

## Highlights

• An optimised pipeline for morphometric analysis of brains with ischaemic damage
• Demonstrate a pattern of cortical atrophy in SVD using VBM and cortical thickness
• The pattern of cortical thinning is distinctive for SVD severity compared to ageing.
• The volume of leucoaraiosis can be predicted from the cortical thickness pattern.

## Graphical abstract

**Figure:**
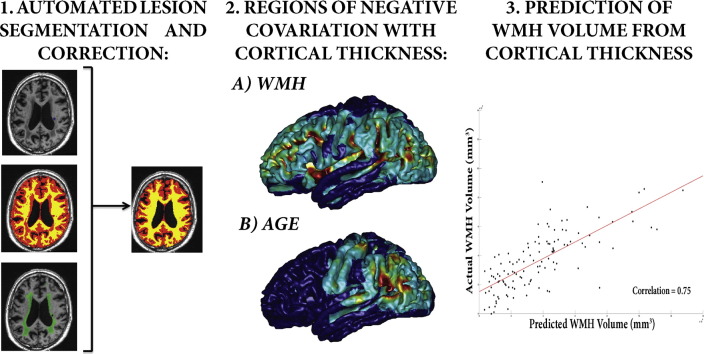

## 1 Introduction

Cerebral small vessel disease (SVD) refers to a heterogeneous group of pathological disorders that, by definition, affect the small vessels of the brain (Pantoni, 2010). They are characterised by typical radiological changes on MRI including white matter hyperintensities (WMH), lacunar infarcts (LI) and cerebral microbleeds (Gouw et al., 2011). It is a highly prevalent disease that increases with age (De Leeuw et al., 2001). SVD is part of a clinical spectrum that ranges from asymptomatic disease through to extensive WMH and LI in symptomatic patients with stroke and vascular dementia (Patel and Markus, 2011; Pantoni, 2010). There is increasing evidence of more subtle morbidities in those with apparently asymptomatic disease. These include cognitive impairment (Lawrence et al., 2013; Pantoni et al., 2007; Prins et al., 2005), gait disturbance (de Laat et al., 2012; de Laat et al., 2010) and depression (Poggesi et al., 2011).

Cognitive impairment in SVD has been shown to associate with a number of different MRI features of SVD including lacunar infarcts, WMH, and less consistently microbleeds (Patel & Markus, 2011); recent evidence suggest that these pathologies are mediated via disruption of complex cortical–subcortical networks (Lawrence et al., 2013). An additional consistent feature associated with cognition impairment in SVD is brain atrophy; whether this occurs due to primary cortical SVD pathology or secondary to white matter changes. Additionally, the mechanism leading to cognitive impairment remains poorly understood. In this study we used a variety of image analysis techniques to further characterise the pattern of cortical atrophy in SVD and define the relationship between grey matter changes and WMH.

### 1.1 Cortical atrophy in cerebral small vessel disease

Whole brain atrophy is a widely reported feature of SVD (Nitkunan et al., 2011; Jokinen et al., 2012), and has been proposed as a simple surrogate of disease progression (Patel and Markus, 2011). However, this measure provides no spatial information regarding the pattern of cortical loss that gives rise to volumetric changes. Furthermore, whole brain atrophy is a feature of a diverse range of potentially overlapping neurological disorders (Fox and Schott, 2004; Sluimer et al., 2010; Schott et al., 2010) as well as in normal ageing (Fjell et al., 2009) and whole brain atrophy measurements fail to distinguish between these different pathologies (Smith et al., 2002). However, characterising the spatial distribution of atrophy may define phenotypic specific patterns (Fox and Schott, 2004), providing a more sensitive and specific means of disease differentiation. Voxel based morphometry (VBM) is a widely used technique that characterises voxel-wise changes in volume within a statistically robust framework (Ashburner and Friston, 2000) and provides insight into spatial patterns that contribute to changes in whole brain volumes (Rohrer et al., 2010). Previous work in SVD using VBM (Raji et al., 2012; Wen et al., 2006) found a correlation between WMH volume (WMHV) and grey matter volume in the dorsolateral prefrontal cortex and posterior portions of the superior and middle temporal gyri. Further evidence for the link between WMH and grey matter changes is found in CADASIL, an autosomal dominant early onset form of SVD, where selective anterior temporal grey matter atrophy has been associated with increasing WMHV (Rossi Espagnet et al., 2012). This work seeks to further these observations by characterising the pattern of GM changes associated with WMHV in sporadic SVD, and differentiate related age associated changes.

### 1.2 Cortical thickness in cerebral small vessel disease

Cortical thickness (CT) studies in SVD are more limited, but may be a promising area, as several pathologies present in SVD would be expected to impact on these measurements. These include disrupted white matter connections due to lacunar infarcts (Duering et al., 2012) or WMH, and the presence of cortical microinfarcts (Smith et al., 2012). Previous attempts to characterise the association between CT and WMHV (van Velsen et al., 2013; de Laat et al., 2012; Reid et al., 2010) in cerebral SVD have yielded few significant results at a global level. However, sub-group analysis by age has indicated a significant negative correlation between WMHV and cortical thickness particularly in the primary auditory cortex (BA 44–45), ventromedial prefrontal cortex (BA10), cingulate gyrus and Broca's area (Reid et al., 2010). The absence of CT changes using whole group analysis techniques may be due, in part, to the region of interest (ROI) based approaches adopted by these previous studies. In particular, these studies averaged CT measurements over automatically parcellated *a priori* regions for which the regional sizes and boundaries have high inter-individual variability when examined histologically (Amunts et al., 2000), resulting in a potential reduction in statistical and spatial sensitivity. Previous studies have also applied surface-based approaches that fit a deformable surface model to an individual brain allowing computation of cortical thickness (Fischl and Dale, 2000). These models rely on accurate surface extraction and have been reliably generated in healthy control subjects. However, difficulties arise in fitting deformed surfaces to deep sulci, buried cortex or abnormally structured brains (Hutton et al., 2008) potentially contributing to the lack of observed findings in SVD using this technique.

Voxel-based cortical thickness (VBCT) (Hutton et al., 2008) is a toolbox implemented in SPM. It uses a voxel-wise layer-growing technique on tissue segmentations in native space to produce a scalar measure of cortical thickness at each voxel location. This enables voxel-wise statistical analysis of cortical thickness using a standard SPM framework and has been previously used to study ageing effects on the cortex (Hutton et al., 2009). This VBCT analysis is a complementary technique to statistical analysis of modulated grey matter in standard VBM analysis, and has been shown to provide a more sensitive measure of healthy age-related grey matter changes (Hutton et al., 2009) due to the fact that cortical folding and surface area have less influence on CT measurements compared to VBM. Whilst it should be noted that voxel-wise methods do not completely circumvent the problems relating to inter-individual anatomical variance at the voxel level due to their reliance on normalisation procedures, the use of newer diffeomorphic-warping techniques (Ashburner and Friston, 2011) with higher anatomical precision (Klein et al., 2009) can allow for less smoothing of the data and therefore improve the sensitivity to detect localised changes in brain structure compared to ROI based approaches.

### 1.3 Machine learning in cerebral small vessel disease

Machine learning techniques are multivariate methods that allow spatial patterns in high dimensional, complex data to be determined. Analysis of these learned patterns is usually performed by classification or regression algorithms with these techniques attempting to generalise or provide predictions from unseen data. Classifiers partition the data into two or more groups and have been clinically used for many tasks such as predicting language outcome after stroke from fMRI data (Saur et al., 2010), diagnosing dementia syndromes (Klöppel et al., 2008) and predicting tumour grade (Zacharaki et al., 2009). Regression methods aim to predict a continuous variable, such as clinical score, from the features within the dataset and have been previously used to predict variables such as age (Franke et al., 2010) and MMSE (Stonnington et al., 2010) from MRI data. Predictive regression methods provide additional anatomical information by determining the spatial features that significantly contribute to the predictions. Here we use a probabilistic regression technique known as Gaussian Process Model Regression (GPR), a Bayesian supervised machine-learning technique for multivariate non-linear regression (Rasmussen, 2006). This technique has been successfully applied to predict recovery of speech function following stroke based on MRI lesion data (Hope et al., 2013). We use it as a complementary multivariate anatomical method to standard univariate VBM and VBCT analyses to better characterise the grey matter correlates of WMH and provide the foundations for future clinical prediction models in SVD.

### 1.4 Hypotheses

This work addresses four hypotheses based on the observations from previous work that, taken together, aim to clarify and define a phenotypic specific pattern of grey matter atrophy (Fox and Schott, 2004) associated with increasing cerebral SVD severity. Our hypotheses are that there is a relationship between SVD severity and volumetric whole brain changes that can be detected using VBM (Raji et al., 2012; Wen et al., 2006). There is a significant inverse association between SVD severity and regional cortical thickness, which can be demonstrated by employing voxel-wise techniques for measuring and analysing cortical thickness measures (Hutton et al., 2008). Furthermore, the pattern of morphometric and cortical thickness changes in SVD is distinct compared to previously demonstrated age related change (Good et al., 2001; Hutton et al., 2009). Finally SVD severity, as quantified by total WMHV, may be predicted from grey matter changes alone using predictive regression techniques.

## 2 Methods

### 2.1 Subjects

121 subjects (78 male, mean age male = 67.96 ± 10 years, female = 73.74 ± 8.12 years) with previously confirmed SVD were recruited as part of the prospective St George's Cognition and Neuroimaging in Stroke (SCANS) study (Lawrence et al., 2013). Recruitment was from acute stroke units or outpatient stroke clinics in three hospitals covering a contiguous catchment area in South London (St George's, King's College and St Thomas' Hospitals). The inclusion criteria comprised a clinical lacunar syndrome (Bamford et al., 1987) with an anatomically corresponding lacunar infarct in addition to evidence of confluent WMH (Fazekas grade ≥ 2) (Fazekas et al., 1987) on MRI. The exclusion criteria were: any cause of stroke mechanism other than SVD, other major central nervous system disorders, major psychiatric disorders, any other cause of white matter disease, contraindications to MRI, or non-fluent in English. All subjects provided written consent and the study was approved by the local ethics committee.

### 2.2 Image acquisition

All of the subject MR images were acquired using the Signa HDxt 1.5 T Magnetic Resonance Scanner (General Electric, Milwaukee, WI, USA) at St George's, University of London. The maximum gradient amplitude was 33 mT m^−1^ and a proprietary 8-channel head coil was used. All subjects were placed in the head coil and an alignment marker was used at the nasal bridge. Velcro straps and foam pads were used where possible to minimise head movement. Whole brain T1-weighted and FLAIR images were acquired for each subject using the following protocol: (1) Fluid Attenuated Inversion Recovery (FLAIR) sequence — TR/TE/TI = 9000/130/2200 ms, field-of-view (FOV) = 240 × 240 mm^2^, matrix = 256 × 192 and 28 axial slices of 5 mm thickness providing a final image resolution of 0.47 × 0.47 × 5 mm^3^. (2) Spoiled gradient echo recalled T1-weighted (SPGR) 3D coronal sequence — TR/TE = 11.5/5 ms, FOV = 240 × 240 mm^2^, matrix = 256 × 192, flip angle = 18° and 176 coronal slices of 1.1 mm thickness providing a final image isotropic resolution of 1.1 mm.

### 2.3 Pre-processing

The raw DICOMS were imported using the SPM8 software package (http://www.fil.ion.ucl.ac.uk/spm/software/spm8/), and each image checked to ensure a common orientation. Initially the T1-weighted and FLAIR images were co-registered together using an affine transformation in SPM for each individual, before affine transformation to the same orientation as the MNI template and resliced to 1 mm isotropic resolution using 4th degree b-spline interpolation.

### 2.4 Segmentation

The conventional SPM segmentation pipelines were adapted and optimised to our study population. This procedure better captures population specific features (such as larger ventricles), allows automated segmentation of a WMH tissue class and allows regions of damaged cortex to be accurately warped to the group average space.

**Fig. 1:**
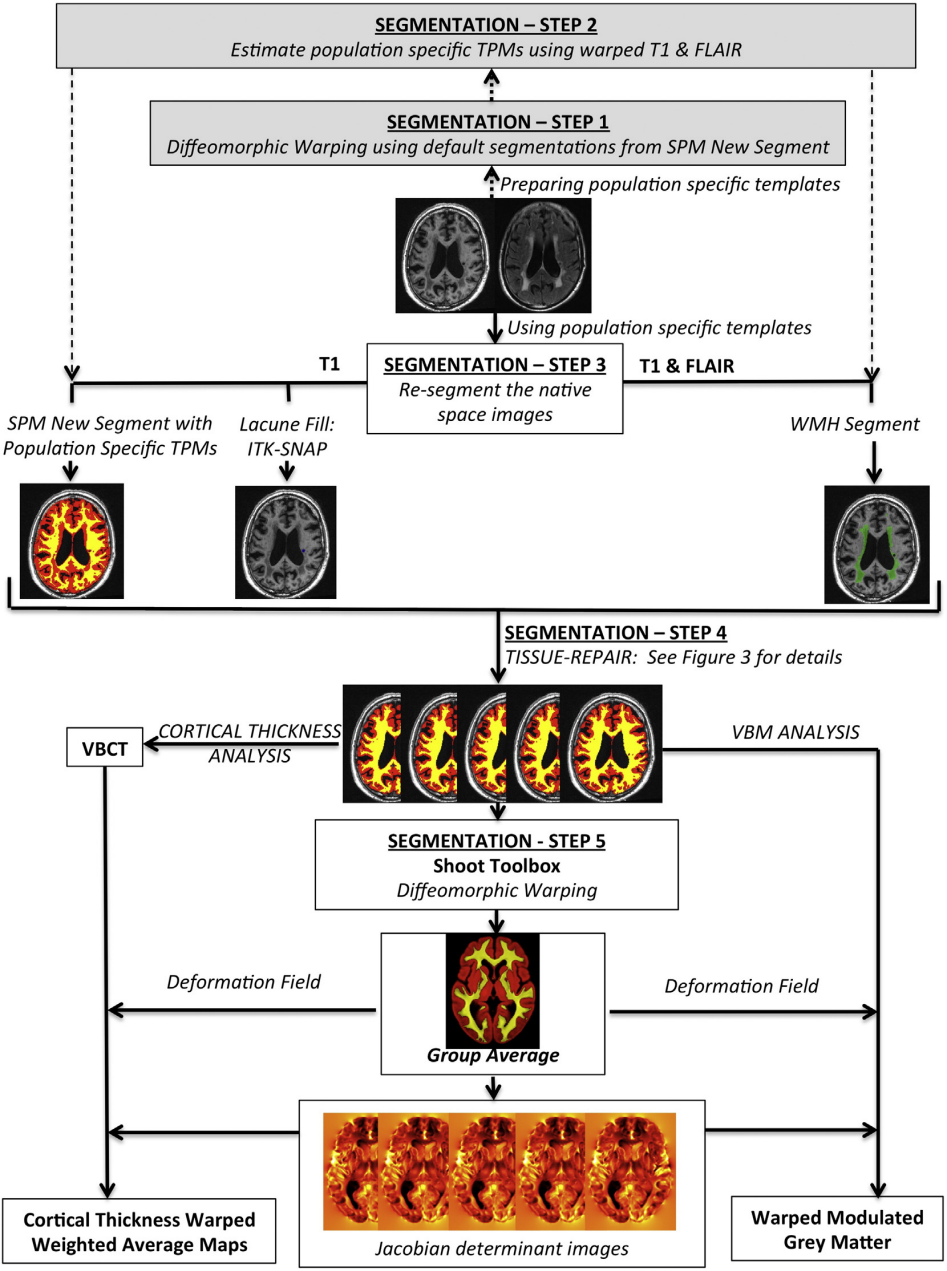
Summary of pre-processing pipeline.

The full pipeline is summarised in Fig. 1 and consisted of five steps. First, a group average template is generated and all the T1-weighted and FLAIR images are warped to this space. Second, the T1-weighted and FLAIR images in the group average space are used to create population specific tissue probability maps (TPMs). Third, these new TPMs are used as *a priori* maps in the SPM segmentation algorithm to re-segment native space images to generate grey matter (GM), white matter (WM), cerebrospinal fluid (CSF) and WMH tissue class segmentation images. These are then combined with manually defined lacune ROIs to provide five tissue class images per individual. Fourth, a tissue repair step is performed and used to repair the GM, WM and CSF maps. This allows an improved estimation of the deformation field to be computed to generate an optimised group average space in the fifth and final step. These steps are further detailed as follows:

#### 2.4.1 Segmentation step 1: generation of group average space

Initially the native space T1-weighted images were segmented into GM, WM and CSF tissue classes using the unified segmentation algorithm within SPM8 (Ashburner and Friston, 2005). These segmentation maps were registered to a group average 1 mm isotropic template using a diffeomorphic registration algorithm (Ashburner and Friston, 2011). The native space T1-weighted and FLAIR images were transformed to this space and skull-stripped using the group average space segmentation images at a probability threshold of 0.1. All skull-stripped images were manually checked and corrected using ITK-SNAP (http://www.itksnap.org) where necessary.

#### 2.4.2 Segmentation step 2: computation of population specific tissue probability maps

Population specific TPMs were computed for GM, WM and CSF from the skull-stripped T1-weighted images in the group average space using a Modified Mixture of Gaussians algorithm to account for cohort specific anatomical features. The skull-stripped FLAIR and T1-weighted images in group average space were then used to generate TPMs for WMH using a Modified Multivariate Mixture of Gaussians algorithm (Lambert et al., 2013). These population specific TPMs then replaced the default *a priori* segmentation maps in the subsequent analysis steps.

#### 2.4.3 Segmentation step 3: re-segmenting the native space images

The population specific TPMs were used to re-segment the native space structural images into GM, WM, CSF and WMH tissue classes using the *New Segment* pipeline (Ashburner and Friston, 2005) in SPM8, with two channels to utilise both the T1-weighted and FLAIR images.

WMH segmentation maps were binarised at a manually determined probability threshold for each individual to ensure that each individual WMH map accurately defined lesion location. Each binary WMH map was visually checked for accuracy and corrected where necessary using ITK-SNAP (Yushkevich et al., 2006; http://www.itksnap.org/). Reliability metrics were calculated as per Dunn et al. (2013). The inter-rater reliability metrics were calculated using the mean values for each rater. WMH reliability metrics were checked using a subset of 20 randomly selected scans by two raters (EZ and CL). As the SCANS study is longitudinal, all potentially available time-points were used for each individual for the calculation of reliability metrics. Both raters were blinded with respect to the subject identity and time point of each scan, and the scan order was randomised for each individual rater to avoid any potential bias. Intra-rater reliability metrics were: Standard error of mean = 247 mm^3^, mean variability = 7.78% (standard deviation = 3.03%) and Pearson's intra-class correlation coefficient = 0.98. The inter-rater reliability metrics were: Standard error of mean = 187 mm^3^, mean variability = 5.57% (standard deviation = 3.99%) and Pearson's intraclass correlation coefficient = 0.99.

Areas of lacunar damage were manually identified on native space T1-weighted images, with reference to the FLAIR images, and segmented using a space-filling algorithm in ITK-SNAP. Grey scale intensity thresholds for the space-filling algorithm were set between 350 and 500 with 500 iterations. Manual refinement was required to ensure accurate representation of LIs adjacent to the lateral ventricles using ITK-SNAP. LI reliability metrics checked using a subset of 20 randomly selected scans by two raters (PB and CL), using the same procedure as the WMH reliability metrics. The intra-rater reliability metrics were: Standard error of mean = 3 mm^3^, mean variability = 7.93% (standard deviation = 4.89%) and Pearson's intra-class correlation coefficient = 0.99. The corresponding inter-rater reliability metrics were: Standard error of mean = 2 mm^3^, mean variability = 4.32% (standard deviation = 4.19%) and Pearson's intraclass correlation coefficient = 0.99.

**Supplementary Fig. 1:**
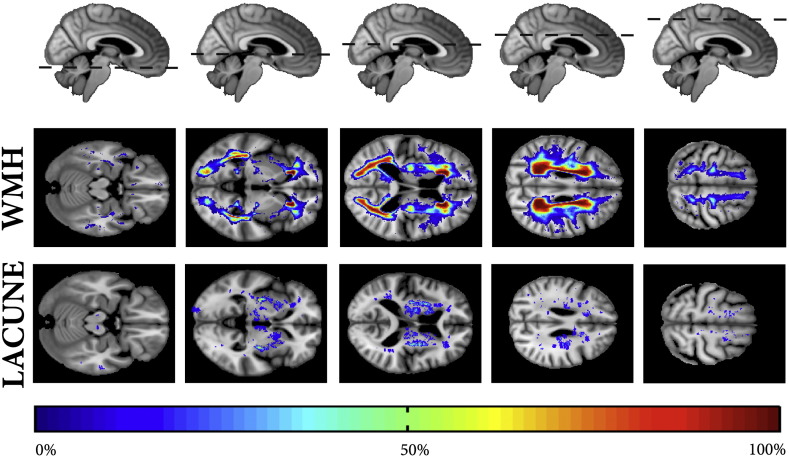
Probability maps projected in group-average space showing the distribution of white matter hyperintensities and lacunar infarcts in the study cohort. Note that the fornix and corpus callosum was excluded from the WMH segmentation by warping a common mask to individual images.

Population WMH and LI distributions are provided in the supplementary data (Supplementary Fig. 1).

#### 2.4.4 Segmentation step 4: tissue repair step

**Fig. 2:**
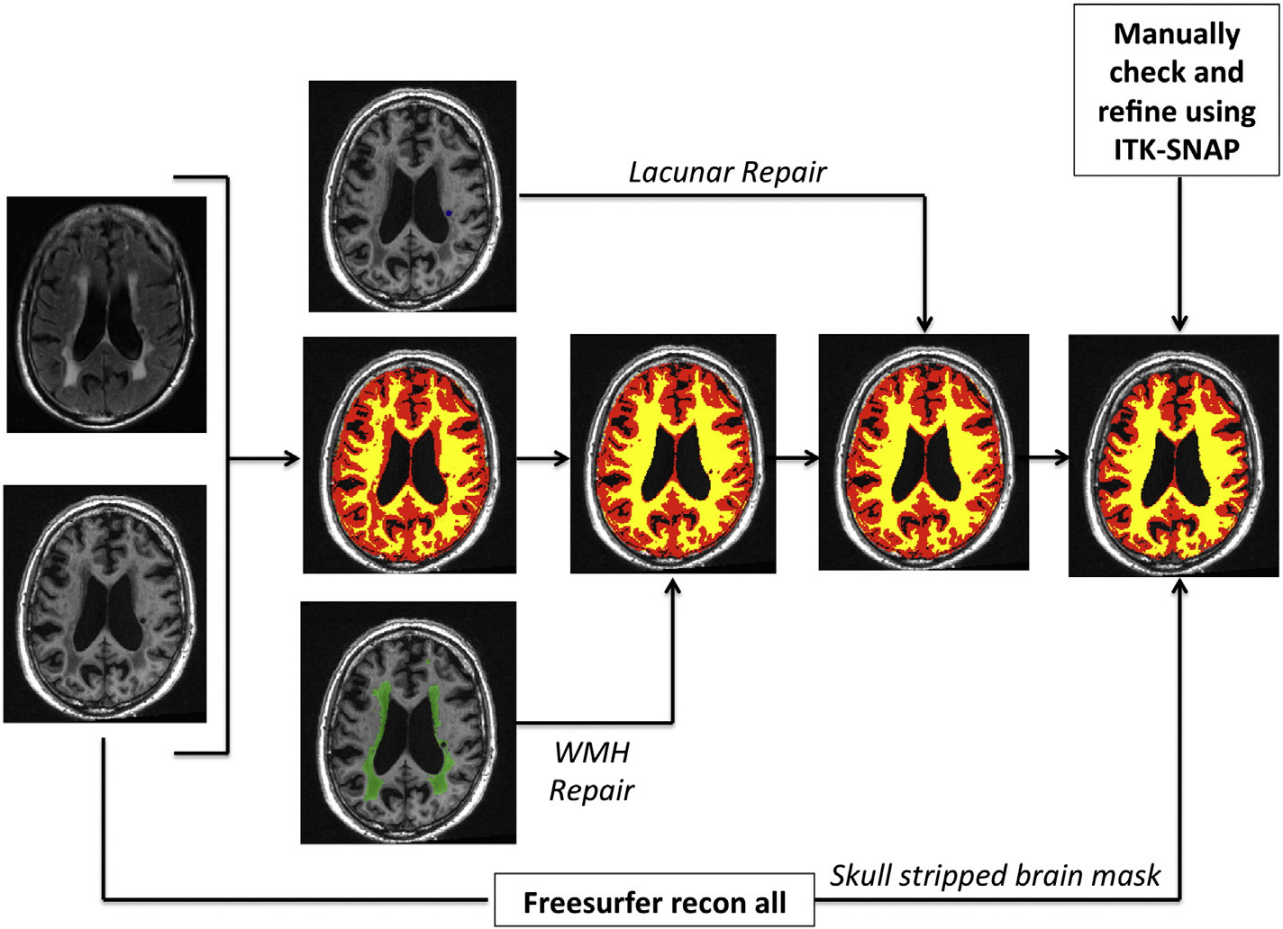
Segmentation repair of tissue misclassification.

A frequent problem with tissue segmentation of brain images of patients with SVD is that regions containing WMH and LI are misclassified into erroneous tissue types due to pathological tissue intensity profiles. This effect is shown in Fig. 2 where regions of WMH are classified as grey matter and gliosis within LI is classified as CSF after application of the unified segmentation algorithm in SPM8. This misclassification causes inaccuracies in computation of the deformation fields required to warp the segmentation images to a group average template and impairs our ability to accurately map regions of damaged tissue to a common space. For example, a region of gliosis caused by a lacune in the head of the caudate would be transformed with the CSF tissue class into the adjacent lateral ventricle. To overcome this problem we developed a segmentation repair step that was carried out in native space. This step is summarised in Fig. 2 and detailed below.

For each individual, the WMH binary maps were used to identify brain regions that should be classed as WM, but had been erroneously classed as GM due to WMH damage. The WMH maps were used to correct the corresponding WM and GM segmentation images. Areas containing LI may be located at tissue boundaries and overlap different tissue classes. To account for this we mapped the binary LI maps to the group average space and classified each LI voxel as GM, WM or CSF depending on the most likely tissue class over the 119 subjects within each voxel of interest. These were then used to reclassify LI voxels within the native GM, WM and CSF maps.

To further improve the segmentations for VBCT, a second skull-stripping step was performed. Each T1-weighted was processed using the recon-all pipeline in Freesurfer (version 5.0, (Fischl et al., 2002; http://freesurfer.net)). The resultant parcellation image was binarised and combined with the repaired segmentations to create an individual brain mask. Each mask was then manually checked in ITK-SNAP to remove any erroneously classified brain voxels around the margins of the skull and dura. These binary mask images were then applied to the repaired GM and WM segmentations. Any removed voxels were added to the CSF segmentation image. The manual segmentation steps were performed by two of the authors (CL and JSN). Ten randomly selected subjects had the segmentation steps repeated to check the consistency between the raters, and a Pearson's intraclass correlation of 0.99 was achieved between the final binarised total brain masks.

Total brain WMHV and LI volume (LIV) in mm^3^ were computed from the binary mask images. Total intracranial volume (TIV) in mm^3^ was calculated from the repaired GM, WM and CSF tissue segmentation maps at a probability threshold ≥0.2.

#### 2.4.5 Segmentation step 5: generation of an optimised group average template

All repaired segmentation maps (Fig. 2) were visually checked for quality in native space and then warped to a final 1 mm isotropic group average template using the diffeomorphic-warping algorithm (the *Shoot* toolbox in SPM12; Ashburner and Friston, 2011)) to generate an optimised group average image for further analysis.

### 2.5 Image analysis

All statistical analyses were performed in MATLAB (2013a) running SPM12 unless otherwise specified. To control for multiple comparisons, Family Wise Error (FWE) correction was used. In SPM, this corrected p value is calculated from the data using Random Field Theory, and is described in detail elsewhere (Nichols, 2012; Worsley et al., 2004). All group analyses were performed in the population group average space. All native repaired grey and white matter segmentations, together with cortical thickness maps were warped to this space using the deformations toolbox in SPM12. Peak Family Wise Error (FWE) p < 0.05 clusters were extracted and Montreal Neurological Institute (MNI) coordinates calculated by co-registering the group average image and thresholded results to MNI space. The Nonlinear Yale MNI to Talairach Conversion Algorithm (Lacadie et al., 2008; http://noodle.med.yale.edu/~papad/mni2tal/) was used to convert the peak coordinates from MNI to Talairach space (Talairach and Tournoux, 1988) to allow the Brede database (Nielsen, 2003; http://hendrix.imm.dtu.dk/services/brededatabase/), to be used to assign the associated anatomical regions.

### 2.6 Voxel based morphometry

The warped repaired grey matter segmentations were modulated by multiplying by the Jacobian determinant, and smoothed using a 6 mm FWHM Gaussian kernel. These were analysed using a multiple regression model in SPM. WMHV, age, gender, LIV and TIV were included as covariates. Regions that survived FWE multiple comparison correction at p < 0.05 were deemed significant. To better explore the FWE significant regions, they are displayed at both p < 0.001 uncorrected and p < 0.05 FWE corrected for multiple comparisons in the figures and appropriate legends provided.

### 2.7 Voxel based cortical thickness

The VBCT toolbox in SPM8 was used to calculate cortical thickness (CT) using the final corrected segmentations. The sampling resolution used was 0.5 mm, CSF smoothness 3 mm, CSF thinness 0.65 and the number of dilations was set to 1. These CT maps were warped to the common final template, and FWHM 6 mm smoothed warped weighted images produced as described by Draganski et al. (2011) (Hutton et al., 2009). The warped-weighted VBCT maps were analysed using a multiple regression model in SPM12 using the same design matrix as the VBM analysis. Statistical significance was set at FWE p < 0.05. As above, regions were visualised at p < 0.001 uncorrected and p < 0.05 FWE corrected for multiple comparisons.

### 2.8 Gaussian process regression SVD prediction

To further investigate the relationship between GM changes and WMH, we attempted to predict WMHV from the grey matter measures alone. Predictive regression was performed using all grey matter voxels across all subjects, using the Gaussian Process Regression algorithm in the PRoNTo toolbox (http://www.mlnl.cs.ucl.ac.uk/pronto/) (Schrouff et al., 2013). PRoNTo includes several kernel-based machine-learning algorithms packaged for use in neuroimaging studies. Typically, the dimension (D) of feature vectors in neuroimaging is the number of voxels available, and these far out-number the actual number of vectors (N), usually the number of subjects. This leads to ill-conditioned problems where D ≫ N. Kernel methods are a powerful collection of algorithms that characterise the pair-wise similarity measures between all examples or patterns in an N × N kernel matrix, and perform the parameter learning using this matrix which avoids issues relating to over-fitting and multicollinearity (Shawe-Taylor and Cristianini, 2004; Hoegaerts et al., 2003; Camp-Valls et al., 2008). Within PRoNTo, the kernel matrix can be further corrected for potential confounding variables, which in our work was age, sex, TIV and LIV. Leave one out cross-validation was applied. Prediction quality was quantitatively assessed by the resulting correlation coefficients and residuals (i.e. the root mean square error between predicted and observed values). Significance was tested using a permutation test (Gollard et al., 2003) set at 1000 repetitions. A final, voxel-wise weight map was calculated and provided in the supplementary results (Supplementary Fig. 3).

## 3 Results

### 3.1 Demographics

121 subjects (76 male) were available for this study, but two were discarded due to severe movement artefact giving 119 for analysis. Mean age at baseline was 70.17 years (range 43.5–88.8). Demographics and vascular risk factors are summarised in Table 1.

Table 2 summarises the MRI measures of SVD severity. The median WMH volume was 32,020 mm^3^ (s.d. ±2590 mm^3^). In our cohort, WMHV did not correlate with age (Pearson's coefficient, r = −0.07, p = 0.47) or total brain volume (Pearson's coefficient, r = 0.13, p = 0.14).

The median lacune infarct volume (LIV) was 263 ± 695 mm^3^. The total LIV correlated with WMHV (Pearson's coefficient, r = 0.37, p < 1 × 10^−4^) so it was also included as a covariate to the general linear model to control for any confounding effect on the grey matter measures. There were no significant gender differences in WMHV or LIV.

### 3.2 Voxel based morphometry

The VBM analysis identified a number of regions of volumetric change associated with WMHV.

**Fig. 3:**
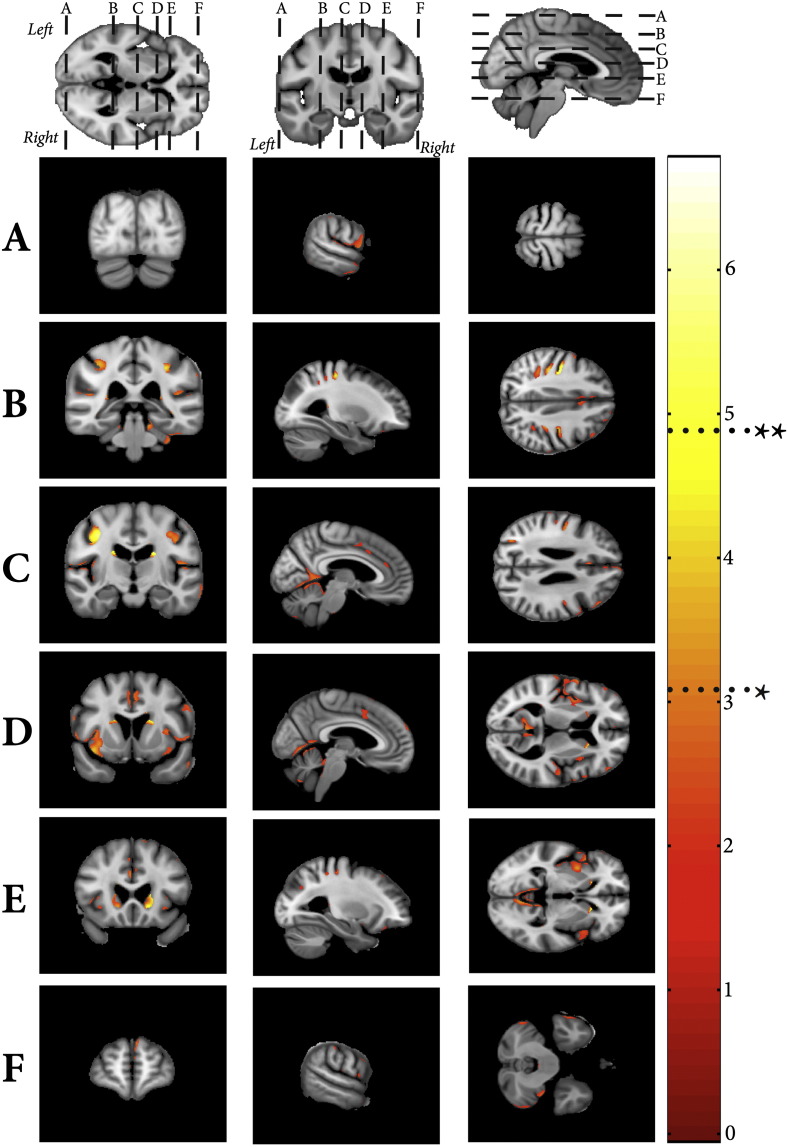
Negative VBM correlates associated with WMH volume. Shown at *p < 0.001 uncorrected and FWE **p < 0.05.

**Fig. 4:**
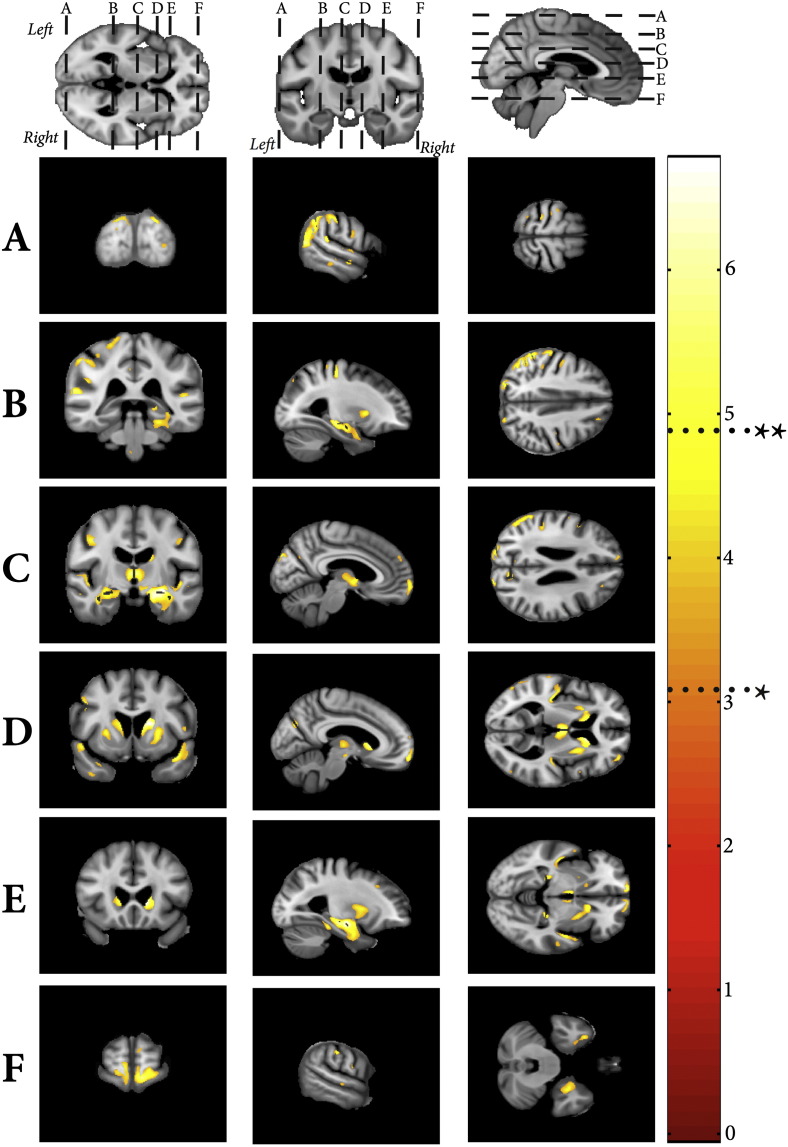
Negative VBM correlates associated with age. Shown at *p < 0.001 uncorrected and FWE **p < 0.05.

Fig. 3 summarises the significant regions of volumetric grey matter loss with increasing WMHV (whilst controlling for age, sex, TIV and LIV). The subcortical regions were the caudate nuclei, affecting both the head and tail bilaterally, in addition to the left putamen and thalamus. Cortically, the main finding was bilateral parietal volume loss, particularly around the supramarginal gyrus and occipital–parietal junction. Detailed information regarding significant regions (including corresponding MNI coordinates) are provided in Supplementary Table 1. In contrast, widespread volumetric loss was associated with age (whilst controlling for WMHV, sex, TIV and LIV), as summarised in Fig. 4. Significant regions include bilateral hippocampus and putamen, anterio-superior temporal gyri, inferior pre-frontal gyri and angular gyri. These ageing findings are consistent with those described by previous authors (Draganski et al., 2011; Smith et al., 2007; Good et al., 2001).

No significant increases in volumetric grey matter were found with increasing WMHV or age.

### 3.3 Cortical thickness

**Fig. 5:**
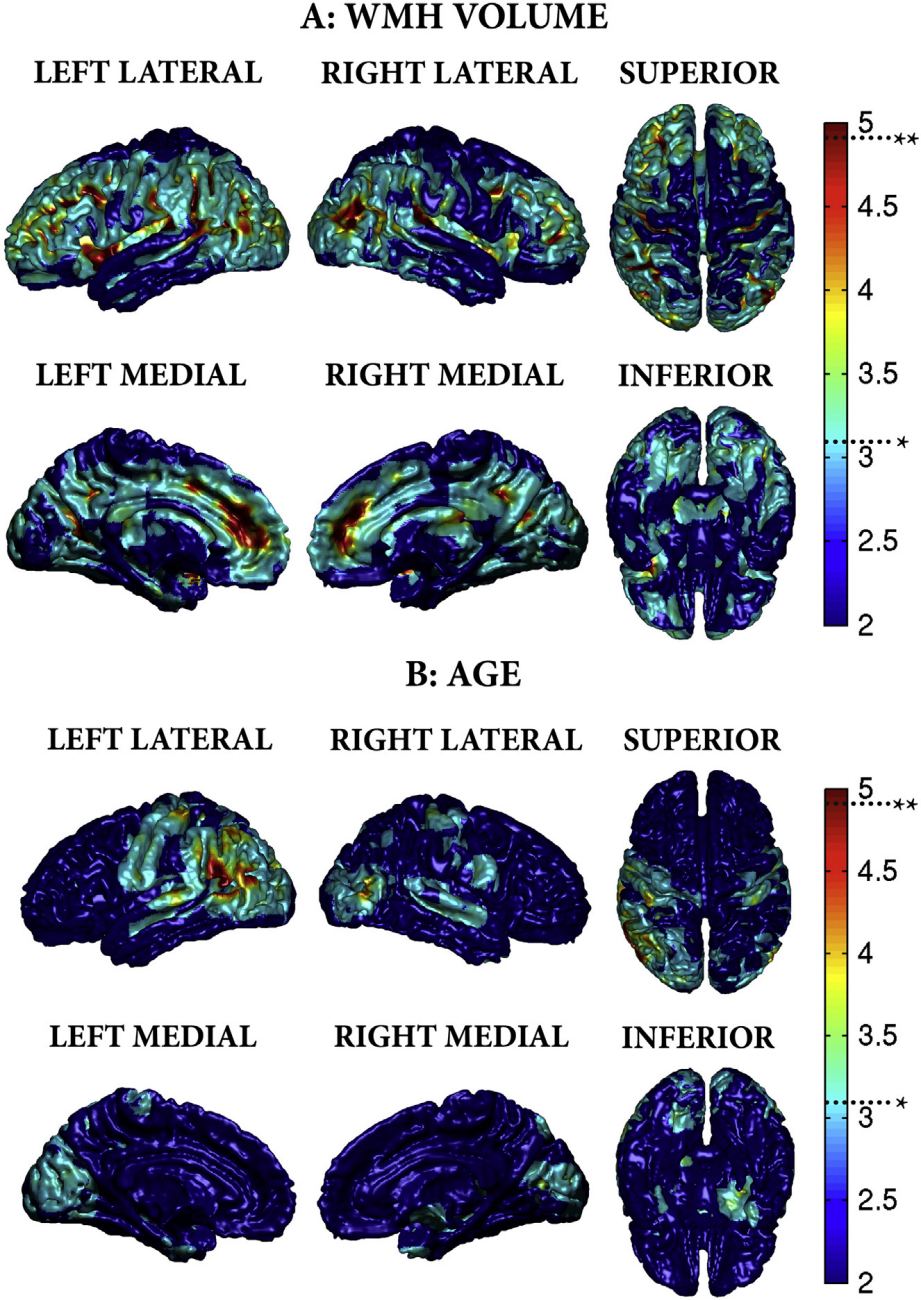
A) Negative VBCT correlates associated with WMH volume. B) Negative VBCT correlates associated with age. Shown at *p < 0.001 uncorrected and FWE **p < 0.05.

Multiple regression analysis did not reveal any regions of cortical thickening with increasing WMHV or age. In contrast, several significant regions which were identified exhibited cortical thinning with increasing WMHV. These regions are summarised in Fig. 5a. Bilateral cortical thinning was found in the insula, dorsolateral frontal, orbitofrontal, cingulate gyrus (particularly anterior) and posterio-inferior parietal cortex. Additionally, posterio-superior temporal lobe changes were located on the medial surface in the lateral fissure in the region of Heschl's gyrus and the auditory cortex. Detailed summaries of these regions with the corresponding MNI coordinates are provided in Supplementary Table 2. In comparison, the age effects whilst controlling for WMHV were characterised by a marked reduction in GM of the pre- and postcentral gyri, temporal gyri (inferior, middle, superior) and angular gyri (Fig. 5b).

### 3.4 Gaussian process regression SVD prediction

**Fig. 6:**
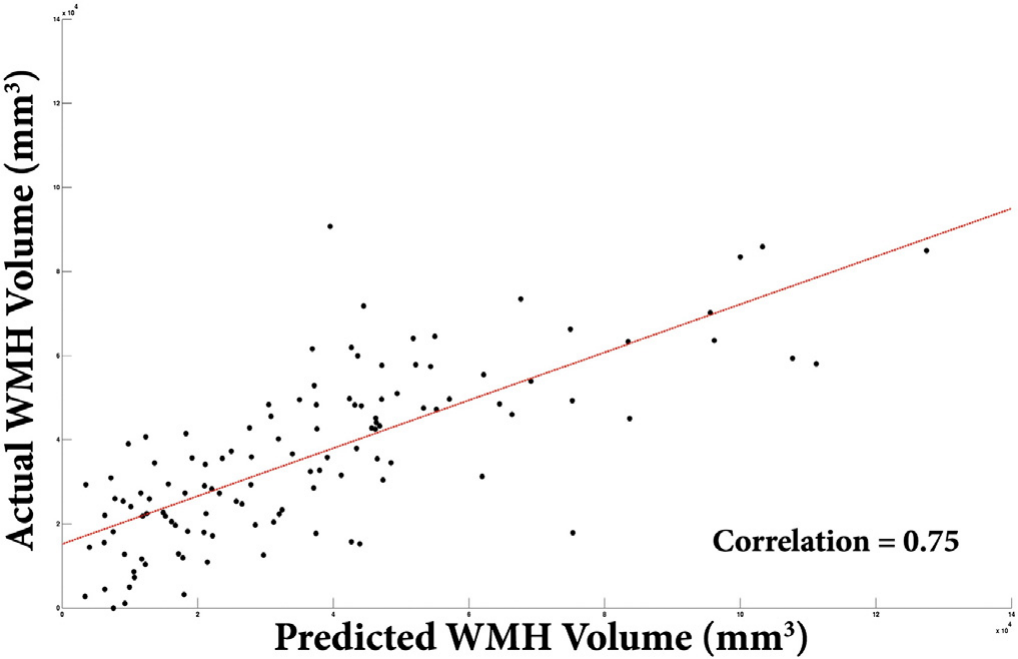
A) Scatter-plot of predicted versus actual WMH volume from cortical thickness measurements.

Significant prediction of WMHV was possible using the modulated grey matter volume and warped weighted cortical thickness maps. Slightly more accurate prediction of WMHV was achieved using the smoothed modulated grey matter (r = 0.80, r^2^ = 0.64, RMSE = 3985 mm^3^) than the cortical thickness maps (r = 0.75, r^2^ = 0.56, RMSE = 4028 mm^3^, see Fig. 6a), presumably due to the addition of subcortical grey matter information in the prediction model. However, this demonstrates that a high degree of prediction accuracy is possible based purely on measurements extracted from the cortical sheet itself.

For completeness, the prediction weight map for the WMH volume and CT GPR has been provided in the supplementary material (Supplementary Fig. 3).

## 4 Discussion

The objective of this study was to define the pattern of grey matter changes associated with SVD, and how they associated with WMH. By utilising a semi-automated, voxel wise technique, a characteristic pattern of grey matter changes associated with increasing WMH volume that is different to age-related effects in grey matter was identified. Furthermore we demonstrated that a quantitative measure of SVD load can be predicted from cortical grey matter measures alone. Taken together, these provide evidence for a regional pattern of cortical atrophy associated with increasing WMH severity in SVD.

### 4.1 Anatomical correlates of WMH severity

Previous studies have examined the VBM (Raji et al., 2012; Wen et al., 2006) and CT (Reid et al., 2010; Tuladhar et al., 2015) correlates of WMH severity; however our current study differs from these in several respects. Specifically, we used an automated pipeline to define WMH volume, producing a continuous volumetric measure of severity, and created optimised population-specific tissue probability maps to improve the quality of tissue segmentations. Additionally, by using an automated tissue repair step with manual refinement to ensure that any misclassified tissue was removed, we further improved the estimations of cortical thickness, deformations and subsequent warping. Finally, the spatial sensitivity of the results was improved by using a newer diffeomorphic-warping algorithm (Geodesic Shooting; Ashburner and Friston, 2011) with higher anatomical precision (Klein et al., 2009) requiring less smoothing of the data (6 mm FWHM as opposed to 12 and 10 mm). Two previous studies are comparable to our approach: Righart and colleagues (Righart et al., 2013) used a 3D spatial cortical thickness model to demonstrate an association between CT and WMH in CADASIL. More recently, Tuladhar et al. (2015) utilised a vertex wide statistical analysis of cortical thickness and a continuous measure of WMH volume. Whilst the pattern of CT atrophy associated with WMH was similar, we demonstrated less temporal lobe involvement, more parieto-occipital atrophy and no regions of cortical expansion. These differences, particularly the latter, may well emerge due to tissue misclassification affecting the CT measurements, as regions of WMH on standard T1 images have a similar intensity profile to GM, and are included in this tissue class when using standard segmentation algorithms (Fig. 2). These errors are more likely to influence CT measurements where there is a high density of WMH lesions close to the cortical mantle, such as the occipital lobe (Supplementary Fig. 1), and any group with a substantial volume of WMH will be particularly susceptible to this. These observations reinforce the importance of accounting for the effects of pathological tissue upon conventional imaging pipelines.

Previous authors have described an association between WMH and age (De Leeuw et al., 2001), which is absent in this cohort. This is likely to be due to one of our inclusion criteria being the presence of confluent WMH resulting in a more homogenous clinical cohort compared to a randomly selected group from the general population.

Despite the highlighted differences, many of our results agree with the broader literature. Our VBM analysis reveals previously reported angular gyrus and insula volumetric reduction with increasing WMH severity (Raji et al., 2012), and novel findings in the caudate and putamen. Previous studies investigating the relationships between CT, WMH and age (Reid et al., 2010) failed to identify any significant correlations between CT and WMH after controlling for age in the whole group analysis. However, they did find an effect in certain Brodmann Area (BA) ROIs using a sub-group analysis when the cohort was split into three age groups, 50–60, 60–70 and over 70. In particular, the strongest effect between WMHV and cortical thinning identified similar regions to those found in our study, including the primary auditory cortex (BA 44–45), ventromedial prefrontal cortex (BA10), cingulate gyrus and Broca's area. In addition to this, they also noted an increase in cortical thickness in BA4 and 5 in the 50–60 and 60–70 age groups respectively which was not observed in this current work. Whilst our result may be partially explained by a higher mean age in our cohort compared to the RUN DMC Cohort (van Norden et al., 2011), the differences in results are more likely due to our application of a voxel-wise approach CT analysis in contrast to computation of average CT within regions of interest. Indeed our CT and VBM changes with ageing, after controlling for WMH volume, are similar to the results of previous studies into normal healthy ageing that used comparable methods (Draganski et al., 2011; Hutton et al., 2009).

We have validated our CT observations by demonstrating that volume of WMH can be predicted from both VBM and CT measurements, and that the removal of subcortical structures only marginally impacts the prediction accuracy, implying a strong association between the cortical sheet and WMHV. Taken together, the VBM, CT and GPR results suggest a phenotypic pattern of cortical atrophy that characterises increasing WMH severity independently of age in sporadic SVD, namely parieto-occipital, posterio-superior temporal, cingulate, middle frontal and orbitofrontal cortical thinning. These overlap, particularly in the frontal regions with previously described grey matter changes in SVD associated with gait (de Laat et al., 2012) and cognitive disturbance (Raji et al., 2012). Furthermore, these regions are distinct from the grey matter changes associated with vascular risk factors such as diabetes (Artero et al., 2004), hypertension (Vuorinen et al., 2013; Seo et al., 2012), hypercholesterolaemia (Krishnadas et al., 2013; Leritz et al., 2011), BMI (Krishnadas et al., 2013; Raji et al., 2010) and smoking (Kühn et al., 2010), suggesting an independent process related to the underlying pathology of WMH.

WMH often begin in the frontal and parietal regions, and increasing WMH severity is reflected by expansion into the occipital and temporal cortices (Artero et al., 2004), together with smooth expansion into the centrum semiovale in the frontal and parietal regions (DeCarli et al., 2005). The underlying histology of MRI WMH lesions in SVD is diverse (Pantoni and Garcia, 1995); myelin rarefaction with axonal destruction is found in the more diffuse centrum semiovale lesions with sparing of the cortico–cortico U-fibres (Revesz et al., 1989). Anatomically, the centrum semiovale is a region that contains several white matter fibre populations: The descending/ascending cortical projection fibres such as the corticospinal tract, the callosal cortico–cortico fibres and long association fibres. However, there is a much higher probability of the anterior–posterior oriented long association fibres passing through regions of WMH (Supplementary Fig. 1), in contrast to the comparatively localised callosal and projection fibre bundles, and hence would be exposed to a greater burden of vascular damage. Conceivably, this damage may result in white matter disconnection, with volumetric reduction occurring in the downstream association cortices. Anatomically, the long association fibres that would be affected and connect the observed regions of cortical atrophy are the cingulum bundle, subcallosal fasciculus, superior longitudinal fasciculus (Türe et al., 2000), arcuate fasciculus, and potentially the superior occipitofrontal fasciculus (Makris et al., 2007). Another factor likely that may contribute to the observed changes are cortical microinfarcts (Schmidtke and Hüll, 2005), which could also cause regional thinning and would be otherwise invisible at standard field strengths (van Veluw et al., 2013). Further work is required to explore and disentangle these findings by clarifying the relationship between longitudinally evolving WMH to quantitative imaging parameters, such as measures of grey matter, diffusion, connectivity and cortical microinfarcts, *in vivo*.

### 4.2 Limitations

There are several limitations with this current work. Whilst every effort was made to develop a fully automated, non-biased approach, the tissue segmentation maps still required a degree of manual correction to accurately remove dura and skull from all participants to provide accurate estimates of CT. Despite this, some of the observed results, particularly in the caudate nuclei, may be influenced by technical difficulties caused by WMH lesions having similar voxel intensity profiles causing tissue misclassification. There was a slight asymmetry in the results, particularly in the age analysis, with more significant results on the left. However, this may be due to differences in cortical variability between hemisphere (Zilles et al., 1997; Mueller et al., 2013), causing interhemispheric differences in image registration. Additionally, the slight skew in results towards sulcal regions may also be due to better image registration occurring in these regions. Both these issues could be resolved in the future by improving image tissue contrast and image resolution, particularly by the acquisition of multimodal imaging data with higher field MR scanners. Higher MR image resolution, using isotropic voxels, would also improve prediction accuracies by providing better estimates of local CT and GM volume due to a reduction in partial volume effects within a voxel.

This work utilised a machine learning technique that had been successfully applied in previous work in a similar cohort (Hope et al., 2013), however, there are several potential techniques available for performing this style of analysis. We investigated the use of alternative techniques including relevance vector machine and kernel ridge regression (Bishop, 2006) but these did not perform as well as GPR in our cohort.

Another issue in performing multivariate analysis is that of multicollinearity, where predictor variables are correlated with one another resulting in a combined prediction of the dependent variable (Kraha et al., 2012). This can lead to models with unstable estimated coefficients and artificially inflated errors; these will impact attempts at statistical inference (Olson-Hunt et al., 2013) and generalise poorly to new data (Rosipal and Trejal, 2001). In the multivariate analysis of whole brain data against a measure of interest, such as a clinical score, this phenomenon will naturally exist due to anatomically connected groups of voxels. However, as in this work, the relationships between all brain voxels (D) over the study population (N) can also be summarised as a kernel matrix of pair-wise similarities (N × N dimensions, instead of N × D), and learning can be performed directly on this matrix. In addition being more computationally efficient (Schrouff et al., 2013), with proper regularisation these kernel methods also enable ill-conditioned problems to be solved that avoid over-fitting and can circumvent issues of multicollinearity (Shawe-Taylor & Cristianini, 2004; Hoegaerts et al., 2003; Camp-Valls et al., 2008). A limitation in these approaches lies in the difficulty in interpreting the final calculated weights or coefficients. This is because it is the combination of all the weights that defines the model, and any attempts to derive spatially specific, statistical inferences will also have to contend with the issues relating to multicollinearity. In this work, we have used GPR to provide supplementary evidence to support the conclusions derived from the univariate VBM and VBCT analyses, and have not attempted to further analyse the weight maps. A future improvement would be to isolate the most informative voxels used by the GPR, rather than having to rely on attempting to interpret the unthresholded, whole-brain pattern (Supplementary Fig. 3). As outlined, locating these voxels is not trivial and is complicated further when there is no strong anatomical *a priori* hypothesis with which to restrict the search space (i.e. which voxels should be removed). Possible solutions would be to place orthogonality constraints on the input data space using principal component analysis or sparse partial least square estimates (Rosipal et al., 2003; Olson-Hunt et al., 2013), or to use Recursive Feature Elimination (Schrouff et al., 2013; De Martino et al., 2008) to iteratively eliminate the least discriminative features based upon the multivariate information itself. However, this latter approach particularly would be extremely computationally intensive to apply to a whole brain analysis (De Martino et al., 2008).

Finally, further methodological improvements may include the use of several data sources simultaneously, such as a combination of cortical thickness and diffusion-weighted MRI data in a single model, using multikernel algorithms that optimise the choice of kernel to improve prediction accuracies (Damoulas & Girolami, 2008).

### 4.3 Future directions

Further work is required to characterise the functional correlates of the observed changes, and define whether physical outcomes can also be predicted from the MRI data alone. This cross-sectional work has demonstrated a tight association between volume of WMH gain and GM loss, both markers of disease severity, however the longitudinal dynamics of these relationships need to be defined. The aim would be to develop these anatomical markers into a longitudinal model to enable individuals with severe or rapidly progressive disease to be identified early, ideally from a single MRI time-point, and quantitatively monitored. Individuals destined to rapid disease progression may well benefit from more aggressive medical therapy started early on. By stratifying groups in this manner, neuroprotective clinical trials could be designed to better identify and optimise the medical management of SVD.

## 5 Conclusion

In this work we demonstrate that WMH severity is associated with regional cortical thinning. Furthermore a quantitative measure of SVD white matter severity as given by WMH volume can be predicted from grey matter measures, supporting an association between white and grey matter damage. The distribution of cortical thinning and volumetric decline is distinctive for WMH severity compared to ageing in this cohort and is consistent with previous studies in this condition. Taken together, these results suggest that there is a phenotypic pattern of atrophy associated with SVD severity.

The following are the supplementary data related to this article.

Supplementary Table 1 Regions of significant (FWE < 0.05) volumetric decline in SVD. Coordinates are given in MNI space. The anatomical region from the nearest coordinate in the Brede database is given.

Supplementary Table 2 Regions of significant (FWE < 0.05) cortical thinning in SVD. Coordinates are given in MNI space. The anatomical region from the nearest coordinate in the Brede database is given.

**Supplementary Fig. 2:**
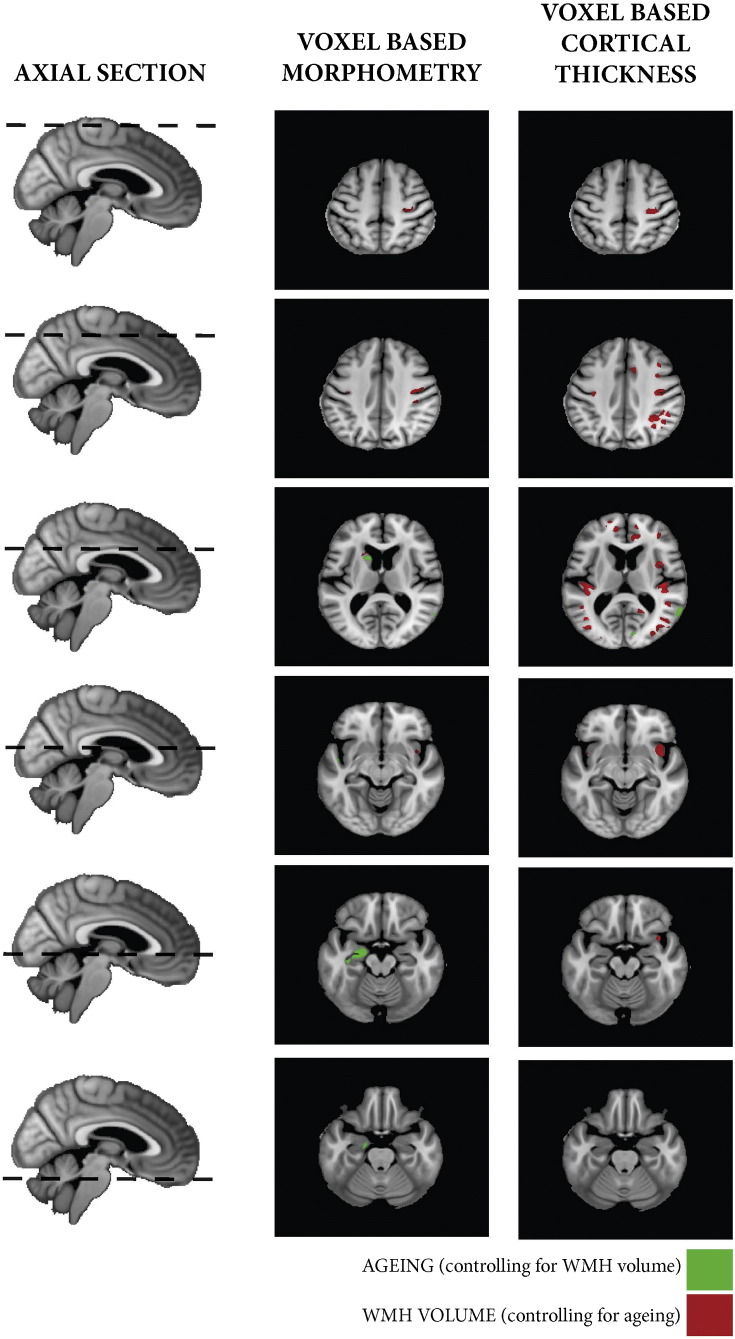
Negative VBM and VBCT correlates binarised at FWE < 0.05. Red — correlates associated with WMH volume. Green — correlates associated with age. Significant regions between the two modalities correspond well, with VBCT being more sensitive to change, except in the subcortical regions that are removed as part of the VBCT analysis (including the hippocampus and caudate).

**Supplementary Fig. 3:**
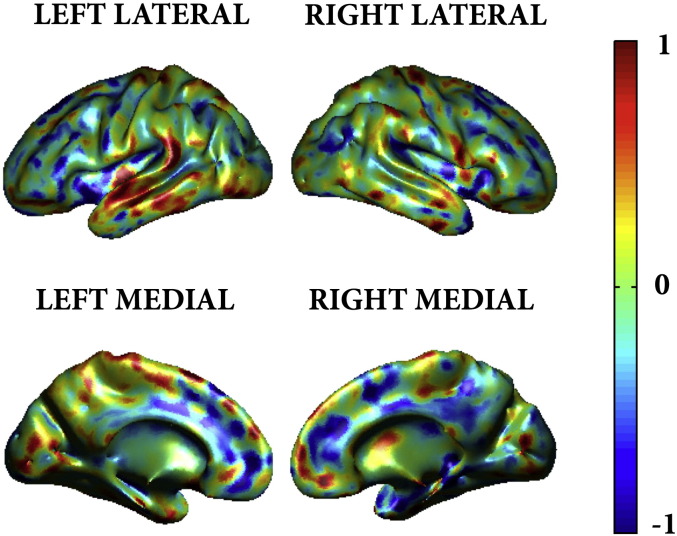
Prediction weights for GPR of WMH volume from cortical thickness projected onto an inflated average brain rendering. Whilst this provides a visual representation as to which cortical regions contributed to the WMHV prediction, the interpretation of this map is not straight forward as individual voxel weights cannot be interpreted in isolation, but rather it is the combination of all the weights that define the model (Schrouff et al., 2013). An in-depth discussion relating to these issues is provided in the limitations.

## Acknowledgements

The SCANS study was supported by a Wellcome Trust grant (0801589). Recruitment was supported by the English National Institute of Health Research (NIHR) Clinical Stroke Research Network. HSM is supported by an NIHR Senior Investigator award. CL is supported by The Academy of Medical Sciences Clinical Lecturer Start-up Grant. Finally we thank all participants from the SCANS study.

## Figures and Tables

**Table 1:** Summary of cohort demographics. Percentages given are as a proportion of the total population used for analysis (N = 119).

DEMOGRAPHICS	N	
***Mean Age in Years (standard deviation)***	***Male (N = 76)***	68.16y (9.81y)
***Female (N = 43)***	73.73y (8.12y)
***Hypertension***		110 (*92%*)
***Treated hypercholesterolaemia***		103 (*87%*)
***Smoker***	***Current***	23 (*20%*)
***Ex***	42 (*35%*)
***Diabetes Mellitus***	***Medically Treated***	21 (*18%*)
***Diet Controlled***	2 (*1.7%*)

**Table 2:** Summary of calculated MRI measures of SVD.

MRI Measure	Mean	Median	Range
***Total WMH Volume (mm^3^)***	36412	32020	3377–127470
***Total Lacune Volume (mm^3^)***	527	263	0–4719
