# Supplementary material for: Characterising the grey matter correlates of leukoaraiosis in cerebral small vessel disease
Source: Neuroimage Clin. 2015 Aug 13;9:194–205. doi: 10.1016/j.nicl.2015.07.002 (PMC4564392; doi:10.1016/j.nicl.2015.07.002)
Supplement: Supplementary Table 1 — Regions of significant (FWE < 0.05) volumetric decline in SVD. Coordinates are given in MNI space. The anatomical region from the nearest coordinate in the Brede database is given. [file mmc1.docx]

|  | **Region** | **MNI Coordinate (mm)** | | | **T-Score** | **Z-Score** |
| --- | --- | --- | --- | --- | --- | --- |
|  |  | **x** | **y** | **z** |  |  |
| **SUBCORTICAL** | Left Putamen | -13 | 19 | 3 | 8.54 | 7.49 |
|  | Left Thalamus | -13 | -17 | 2 | 7.4 | 6.67 |
|  | Left Caudate Nucleus (Tail) | -17 | -19 | 23 | 7.32 | 6.61 |
|  | Left Caudate Nucleus | -13 | 12 | 17 | 5.05 | 4.78 |
|  | Right Caudate Nucleus | 11 | 19 | 2 | 7.16 | 6.49 |
|  | Right Caudate Nucleus | 13 | 14 | 16 | 6.92 | 6.31 |
|  | Right Caudate Nucleus (body) | 13 | 11 | 18 | 6.87 | 6.27 |
|  | Right Caudate Nucleus (head) | 13 | 4 | 22 | 5.65 | 5.29 |
| **INSULA** | Left anterior insula | -31 | -1 | 0 | 5.9 | 5.5 |
| **PARIETAL** | Left primary sensory cortex | -32 | -18 | 35 | 8.13 | 7.21 |
|  | Left supramarginal gyrus | -32 | -32 | 35 | 6.03 | 5.6 |
|  | Right posterior parietal | 31 | -36 | 38 | 6.04 | 5.62 |
|  | Right inferior parietal lobule | 42 | -14 | 29 | 5.97 | 5.26 |
|  | Right precuneus | 7 | -66 | 3 | 5.23 | 4.94 |
| **OCCIPITAL** | Left middle occipital gyrus | -29 | -73 | 24 | 5.11 | 4.84 |
| **TEMPORAL** | Right superior temporal gyrus | 21 | -31 | 19 | 9.75 | Inf |
| **CINGULATE** | Left cingulate gyrus | -13 | 2 | 22 | 5 | 4.75 |

*Supplementary table 1: Regions of significant (FWE<0.05) volumetric decline in SVD. Coordinates are given in MNI space. The anatomical region from the nearest coordinate in the Brede database is given.*
