# Supplementary material for: Characterising the grey matter correlates of leukoaraiosis in cerebral small vessel disease
Source: Neuroimage Clin. 2015 Aug 13;9:194–205. doi: 10.1016/j.nicl.2015.07.002 (PMC4564392; doi:10.1016/j.nicl.2015.07.002)
Supplement: Supplementary Table 2 — Regions of significant (FWE < 0.05) cortical thinning in SVD. Coordinates are given in MNI space. The anatomical region from the nearest coordinate in the Brede database is given. [file mmc2.docx]

|  | **Region** | **MNI Coordinate (mm)** | | | **T-Score** | **Z-Score** |
| --- | --- | --- | --- | --- | --- | --- |
|  |  | **x** | **y** | **z** |  |  |
| **INSULA** | Left Anterior Insula | -29 | 23 | 20 | 7.28 | 6.58 |
|  | Right Anterior Insula | 39 | -3 | -2 | 5.2 | 4.92 |
| **FRONTAL** | Left Precentral Gyrus | -32 | -1 | 34 | 5.55 | 5.21 |
|  | Left Precentral Gyrus | -27 | -1 | 36 | 5.36 | 5.05 |
|  | Right Prefrontal Cortex | 31 | 37 | 5 | 5.76 | 5.38 |
|  | Right Middle Frontal Gyrus | 33 | 6 | 30 | 5.63 | 5.28 |
|  | Right Medial Frontal Gyrus | 11 | 46 | -2 | 5.33 | 5.03 |
|  | Right Orbitofrontal gyrus | 15 | 31 | -3 | 5.2 | 4.91 |
| **PARIETAL** | Left Supramarginal Gyrus | -21 | -41 | 24 | 6.72 | 6.15 |
|  | Left Precuneus | -11 | -58 | 35 | 5.71 | 5.34 |
|  | Left Deep Inferior Parietal | -44 | -22 | 28 | 5.67 | 5.31 |
|  | Right Supramarginal Gyrus | 32 | -22 | 34 | 6.06 | 5.63 |
|  | Right Precuneus | 14 | -56 | 17 | 5.34 | 5.04 |
|  | Right Supramarginal Gyrus | 47 | -43 | 33 | 5.29 | 4.99 |
|  | Right Supramarginal Gyrus | 48 | -18 | 21 | 5.23 | 4.94 |
|  | Right Inferior Parietal Cortex | 46 | -48 | 29 | 5.06 | 4.8 |
| **OCCIPITAL** | Left Lingual | -31 | -60 | 0 | 5.29 | 4.99 |
|  | Right Occipital (Cuneus) | 16 | -72 | 6 | 5.25 | 4.96 |
| **TEMPORAL** | Left Superior Temporal Gyrus | -41 | -48 | 19 | 6.92 | 6.31 |
|  | Left Superior Temporal Gyrus | -44 | -50 | 3 | 5.25 | 4.96 |
|  | Left Medial Temporal Lobe | -39 | -11 | -15 | 5.15 | 4.87 |
|  | Right Posterior Fusiform | 39 | -66 | 12 | 7.64 | 6.85 |
|  | Right Posterior Middle Temporal Lobe | 36 | -61 | 29 | 6.27 | 5.8 |
|  | Right Superior Temporal Gyrus | 21 | -33 | 19 | 5.77 | 5.39 |
|  | Right Middle Temporal Gyrus | 29 | -53 | 18 | 5.28 | 4.98 |
|  | Right Middle Temporal Gyrus | 45 | -52 | 3 | 5.15 | 4.87 |
|  | Right Superior Temporal Gyrus (Near Heschl's) | 47 | -25 | 7 | 5.11 | 4.84 |
| **CINGULATE** | Left Anterior Cingulate | -15 | -9 | 39 | 5.56 | 5.22 |
|  | Left Middle Cingulate | -7 | 6 | 33 | 5.46 | 5.13 |
|  | Right Posterior Cingulate | 11 | -50 | 27 | 5.97 | 5.55 |
|  | Right Middle Cingulate | 21 | -8 | 26 | 5.61 | 5.26 |

*Supplementary table 2: Regions of significant (FWE<0.05) cortical thinning in SVD. Coordinates are given in MNI space. The anatomical region from the nearest coordinate in the Brede database is given.*
